# The Blows and Capoeira Movements From the Caricatures of Calixto Cordeiro

**DOI:** 10.3389/fpsyg.2020.586712

**Published:** 2020-10-28

**Authors:** Paulo Coêlho Araújo, Ana Rosa Jaqueira

**Affiliations:** Faculty of Sport Sciences and Physical Education, University of Coimbra, Coimbra, Portugal

**Keywords:** capoeira, caricature, strikes, history, Brazil, iconography, iconology

## Abstract

This study analyses some elements of the historical and social nature of Capoeira, such as the juridical-political, the clothing, the symbolic and the identity, but, essentially, those of a physical and linguistic nature, from the caricatures produced by Calixto Cordeiro, which illustrated the work of Lima Campos in 1906. In this work, a multi-method process was applied, represented by the presuppositions of Historical Archeology, the interpretation of images indicated by Panofsky, and the documental analysis of various sources. The use of these presuppositions and the confrontation of sources of diverse natures allowed us to interpret the signs, symbols, and meanings from the facts and data described by Cordeiro, about Capoeira presented by different literates. The results of these analyses showed substantial information about the blows/moves of this struggle, as well as its different names, slang and expressive forms, often associated with different groups of practitioners from different Brazilian cities.

## Introduction

The interest in this approach to image interpretation attributed directly or even indirectly to the fight/game of Capoeira comes from several distinct factors, from which we emphasize the virtual inexistence of any imagery analysis of the mentioned fight/game, coupled with the fact that we can identify in its specific literature superficial and little informed interpretations of some of the iconography that portrayed the presence of this expression in the Brazilian society of the 19th and 20th centuries.

By choosing the iconographic interpretation as part of the historical and social analysis of Capoeira, we use the concepts of Panofsky (1986) on iconography, which is:

*The branch of Art History that deals with the subject content or meaning of works of art as something different from its form, and according to the theory of iconicity is the iconography (…) the rewriting of reality, comprising a whole universe of nonverbal signs of figurative manifestation ranging from drawing to photography* (Panofsky, 1986: p. 19)

Despite identifying a growing number of studies on the interpretation of various types of images, more specifically of images produced by different artists and the most diverse techniques over the 18th and 19th centuries in Brazil, we recognize that there are still only a few studies produced by scholars from the various fields of knowledge addressing Capoeira as the analytical focus that allows us to interpret the signs and meanings, the facts, and data presented in the traces of artists of yesteryear.

Recognizing that there is a gap in the context of iconographic interpretations of Capoeira, and identifying a significant volume of images that portrayed aspects of Brazilian colonial, imperial and republican customs, there highlighting bodily expressions of fight that would bring us closer to the consideration of being these, the fight/game of Capoeira – we take it as necessary to venture out in the analysis of the intricacies of such records, thus allowing ourselves to confirm the icons represented by the artists in their most distinct forms of artistic expression (Araújo and Jaqueira, 2017), shedding light on some aspects of the history of this genuinely national body art.

The study now elected stems from the need to record new scientific approaches developed for the Brazilian fight in order to promote further analysis on this body expression in various aspects, and in this case, on the presence and evolution of the strikes/body movements in the context of Capoeira. This work is scientifically based on the theoretical framework of Historical Archeology in order to, through selected images, contribute through the highlighted icons to a more complete understanding and reconstruction of this fight and aspects of daily life of practitioners of Capoeira and the resulting dynamics occurring in its context.

## The Scientific Basis of the Study

Considering the richness of information contained in an image, and the different elements that can be extracted from it, we believe that a single methodological procedure would not allow us to cover the different nuances present in the image that we analyze here. Starting from this observation and the scarcity of iconographic and iconological analysis in the context of Capoeira, we consider that a multi-methodological framework would allow us to validate the different elements of a juridical-political nature, the clothing, the symbolic and identity, and mainly, those of a bodily and linguistic nature, as well as the descriptions of the authors associated with the images appreciated.

We recognize, therefore, that the use of a multi-method procedure will allow us to fill gaps derived from the insufficiency of each of the assumptions applied here for the analysis in question and to extract as much information as possible about the object of analysis and the social reality where it manifested itself, reconstructing and reinterpreting according to Orser (1992), “repressed cultures, forgotten practices, suffocated voices,” and thus the material and immaterial culture of the illiterate.

These methodological assumptions of Historical Archeology (Orser, 1992), of interpretation of Panofsky (1986), and of the documental and bibliographical analysis allowed us to reconstitute aspects of the daily life of Capoeira in the 19th and early 20th centuries, considering for analysis the elements of a documental nature compulsed in Brazilian archives and libraries and bibliographical literature on Capoeira for this temporal space.

The multi-methodological foundations that supported this interpretation, first iconographic and later iconological, for promoting a break with the discourses of common sense, were those inherent to that of Historical Archeology, that of bibliographic and documentary analysis for its abundance and richness of sources on this Brazilian expression, and that of determinants for this iconographic/iconological interpretation, the assumptions presented by Panofsky about the interpretation of images, mainly because they are scarce for the context of Capoeira, interpretations of images supported by a theoretical assumption that proved applicable for the intended analysis in this specific case and for a set of previously selected images.

This methodological framework was thus structured for better application to this type of work in particular for presenting characteristics that allow, in a more flexible way, to select techniques that can lead to an extensive and varied data collection, allowing the clarification or a better understanding of the selected object (Capoeira), either by the quantity or the quality of the material collected in view of the interaction of all elements, material, non-material, and human present in a historical period, and the consequent influence on the behavior of individuals identified as *capoeiras*.^1^

This multidisciplinary approach allows us, through the different sources used, on the one hand, a wider understanding of society and its manifestations, and on the other hand, the reconstruction of aspects of the reality closer to the daily life of those who practice Capoeira fighting/playing in Brazilian historical periods.

The assumptions of a field of study called Historical Archeology emphasizes that there is no scientific predominance of one area over another and that it is necessary to be able to understand it as a diversified and multidisciplinary field of approaches that combine correlated areas, thus promoting “a partnership between men and things” (Orser, 1992), and that contribute to the achievement of their own objectives.

In face of the multidisciplinary character of this field, approaches and appraisals from different sources are highlighted for each specific field of study, with elements of a cultural nature such as “oral testimonies, ethnographic records, folklore”^2^ (Orser, 1992), for the field of History, “historical documents,” and for the field of Archeology, “historical architecture, artifacts” and structures, and also pictorial elements, where one can find paintings and drawings in their most in its various expressions, and photographs as well. Regarding the typology of the sources consulted, among those presented to us in the field of Historical Archeology, we highlight those translated into pictorial information and expressed by drawings, paintings, caricatures, and photographs (Orser, 1992).

For this study, we selected six caricatures produced in 1906 by the Rio artist Calixto Cordeiro (1877/1957), an artist who played several different roles throughout his life. He stood out both as a writer and a playwright, as an illustrator, as a sculptor, and as a poet, among others, and contributed to numerous magazines and newspapers of his time. The selected caricatures deal with the fight of Capoeira and particularly with some structural elements of Capoeira, which complement the textual analyses elaborated by Lima Campos^3^ and “indicate some of the most common defense and attack moves and provide an idea of the technical slang.”^4^

To support the analyses, we resort to a considerable body of primary (Brasil Arquivo Nacional, 1920), and secondary sources produced by distinguished characters during the 19th and 20th centuries, about the history of Brazil in general, and Capoeira in particular, where written documents stand out, as well as travel records, diverse legislation, oral information, diverse iconography, and several others, and also those that require analysis and interpretation of contemporary literary works that have published information about this representation of Brazilian fight.

From the analysis of the selected images that portrayed aspects of the Capoeira fight in the early years of the 20th century, we consider the need for understanding a particular fact, and for that, we turn to the concepts formulated by Alarcão (2000) which tell us that fact “is what was, what has gone by, that cannot be observed in the present,”^5^ or a specific datum, which the same author considers as a “trace, being what one finds that is there, before my eyes, but also that from which something else is deduced, that is no longer observable. The datum is what is.”^6^

Sustained on the scientific arguments presented by Panofsky on the interpretation of images and included in the introduction to his book Iconology Studies (1986), we present his summary table that will allow us to demonstrate the possibilities of interpretation of the selected iconography about Capoeira, identifying from concepts presented by Alarcão (2000), what would fit in them as data or facts, making us infer being such images representations of the manifestation called in other times and at present, as the Brazilian game/fight of Capoeira.

Based on the assumptions indicated by this author with regard to the interpretation of images, the consideration of the object, the act, and the baggage of interpretation as fundamental elements to extract the data and facts derived from the images under analysis, indicating to the researcher the appropriate procedures for the promotion of a consequent analysis, portrays through the combination of other information, a history of these men and their cultural practices, which go beyond the simple iconographic interpretation and, thus, promote an iconological interpretation that goes deeper than the simple description of the pictorial element analyzed.

Of the three items of interpretations highlighted, object, act, and background knowledge (Table 1), some aspects arise, the ones that have allowed us to conduct our iconographic analyses and, thus, enable us to make inferences about the Capoeira expression, which over time have not been fruitful in discourses that could allow us to state categorically its presence in all known iconic representations.

**Table 1:** Adaptation of Panofsky’s Synoptic Table on image interpretation.

**Object of interpretation**	Primary or natural subject content (factual, expressive) constituting the world of artistic motives	Secondary thematic content, constituting the world of images, stories and allegories	Intrinsic meaning or content, which is the world of values
**Act of interpretation**	Pre-iconographic description (and pseudoformal analysis)	Iconographic analysis, in the strictest sense of the word	Iconographic interpretation, in the deepest sense (iconographic synthesis)
**Background knowledge for interpretation**	Practical experience (familiarity with objects and actions)	Knowledge of literary sources (familiarity with specific themes and concepts)	Synthetic intuition (familiarity with the essential tendencies of the human spirit conditioned by psychology

From the data present in caricatures under focus, we cannot afford to understand them as the true story of marginal groups of Brazilian society depicted there but as an extract of the social reality presented from the perspective of the author and complemented by the considerations of Lima Campos over a certain group of individuals of this society, transferring its own ideology, values, and specific knowledge about structural elements of Capoeira.

It is necessary to reinforce the idea that the iconographies do not represent the real truth of events, expressivities, and historical objects that the author narrates as if they had happened, but his perception and all the appropriations of the society he lived in, which are confirmed by the obtained data sets of the data collected in the different sources consulted. The images selected in this work are as visual certificates of a past event, experienced, imagined, that is only embodied as a possible temporal truth, becoming real with content and information from other related fields and a series of documentary media, written and oral.

The axis of this reflection aims at probing the cultural history of Capoeira through its imagistic representations in the time and space referred to, bearing in mind all the social, political, legal, and law-enforcement variables, among others, in the ambient that allowed the author of the images the reliable reproduction of the observed reality, although they may include general observations of his social perception, highlighted by their “features, aspects, symbols, representations, hidden dimensions, perspectives, inductions, codes, colors and shapes present in them.”^7^

We also call the attention to the care that we went through when analyzing such images, given the fact that the specific literature of Capoeira often includes bizarre statements about certain signs present in its context, that remain to this day as if they were truths, considering contemporary assessments of this form of expression, but in the past, perhaps, did not have the representation attributed to them. I, therefore, corroborate the statements of Paiva (2002) in which,

*We must always be aware of the existing limitations of these interpretive procedures, failing which, in the extreme, of inventing historical realities in order to adapt them to the audited iconography. Or, which is as pernicious as the previous situation, of inventing meanings to better fit an image in its time. Or even still, taking to the past and to the iconic representation values of our time, which did not exist before, which consists of anachronism, the greatest sin, so to speak, that a historian can come to commit.* ^8^

It is impossible today to deny the importance of image interpretation in producing innovative work to recount the history of Brazil and, therefore, its distinct human groups and their manifestations, interpretations which, more present in the works of Social and Human Sciences, and virtually inexistent within the Sports and Physical Education Sciences, in particular in the distinctive fields of expression of Capoeira, are mostly due to the unconditional acceptance of the positions of interpreters from other areas, who often use the common sense discourses of practitioners of this Brazilian fight.

To analyze aspects relating to data and facts of an iconography about Capoeira, from the interpretation of the processes listed by Panosfski, we chose the six caricatures produced by Calixto Cordeiro to illustrate the text of Lima Campos (1906), named after the order in which they are presented in his work as: types and uniform; The Sifting; The Coconut Strike; The Shim or Tripping; The Oil Lamp; and Tuck the Move, so thus promoting comments that contribute to a better understanding of its own signs, symbols, and representations and the positions of the authors or third parties, assumed in the interpretation of these iconographies.

## The Caricatures of Calixto Cordeiro and Capoeira: Historical Considerations Headings

The caricatures of Calixto Cordeiro were elaborated to underpin Lima Campos’s text entitled Capoeira,^9^ whose theme was the presentation of the components of Capoeira fight in Rio de Janeiro in the carioca (Rio inhabitant) first republic context. These caricatures identify some of the elements in the context of this sport, especially references on movements and strikes, the technical slang, very common among practitioners of this body language during the 19th and early 20th centuries, and some distinctive features between groups^10^ of *capoeiras* of that period.

The distinctive features took into account the presence of *Maltas*,^11^ *Nagoas*, and *Guayamús* in Rio de Janeiro, considering the allusions to their colors and the way of using the hat – for the former, the strap with white on red and the hat with a front brim pointing forward, and for the latter, strap with red on white and a hat with a front brim lifted upwards.

The reasons portrayed in Calixto Cordeiro’s caricatures allowed Lima Campos to evince his deep knowledge about the foul language used by practitioners of Carioca Capoeira in the first decade of the 20th century, as well as words only used in popular contexts and only understood by its visitors. From this author’s texts, we draw words like *cueré-réca*, *churumella*, *estrompício*, *marchante*, *sarado*, *joça*, *figuração*, *chinxa*, *caveira*, *turuna, cutuba*, many of these terms not appearing in current dictionaries, but that were commonplace in the statements of the regulars of trickery groups and by practitioners of Capoeira from this period.

The caricatured and accompanied images of the respective names of the movements or strikes used in Capoeira in Rio de Janeiro in the early decades of the 20th century, remarkably The Sifting, The Coconut Strike, The Shim or Tripping, The Oil Lamp, and Tuck the Move, allow us to clearly identify the characteristic of the movement, since the description by Lima Campos in his text, in some cases, could only be understood by the regulars of the Rio bohemian environment, for its popular use and for its foul and coded language.

Lima Campos, author of the paper entitled “A Capoeira”, besides highlighting his impression and knowledge about Capoeira, refers to the caricatures of Calixto Cordeiro, as indicative of some of the most common defense and attack strikes and give some insight on technical slang, and in our view, promote as well a unique way of preserving the body memory of Capoeira and the linguistic memory of the practitioners of the Brazilian fight in a given period of time, if we consider the documentary lack of any nature in former periods that mentioned these cultural elements.

The records of body memory of Capoeira were treated episodically by Rugendas (1940), Earle (quoted in James, 1955), and Cristhiano Junior (quoted in Leite, 2002), while the linguistic and descriptive records of body shapes identified by strikes/movements were only highlighted by ODC (1907), Edmundo (1932) and Moraes Filho (1999) by some handwritten documents from police authorities of the early 19th century and others who repeated the allusions of these authors without any insights on these and other movements and the language used in the context of the Brazilian fight.

Of the six caricatures presented by Calixto Cordeiro and aimed at a more conscious and objective methodology of work, we divided them into two types of features that will provide the elements of analysis, in which gain relevance, first, the costumes of the caricatured elements, followed by the presentation of some of the specific materials and movements of Capoeira, very usual during the 20th century.

As far as the costumes of the individuals depicted are concerned, we can even claim that the ones shown in the pictures are very different from those that have historically been assigned to these characters, either in Rio de Janeiro or in Bahia, and even to those listed in the literature about this fight, which are founded solely on oral tradition and have no evidence to support them.

The iconographic representations of Calixto Cordeiro concerning Types and Uniforms subsidize the descriptions of Lima Campos by showing the colors of the costumes of Rio’s gangs and the use of the hat placed the right way while still allowing Lima Campos to extract from these caricatures other common components by the mentioned *capoeiras*, such as plaid tie and slip ring and nozzle boots, pieces also described by Moraes Filho,^12^ and also highlighted in the image named *Furdunço* by Álvaro Martins (quoted in Bretas, 1989).

About the kinds, and contrary to the speeches of advocates that Capoeira was practised in the 19th and early 20th centuries exclusively by Black people, the representations of the artist showed the presence of Blacks and Mulattos, well typified in the various caricatures presented, specifically in the caricature Types and Uniforms, also represented by Álvaro Martins (quoted in Santos, n.d.) in the drawing identified as *Furdunço* (Bretas, 1989).

**Figure 1:**
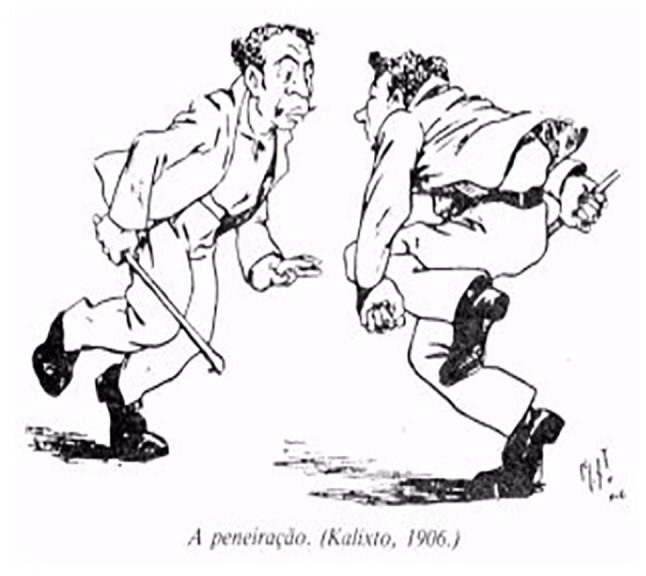
Types and uniforms.

**Figure 2:**
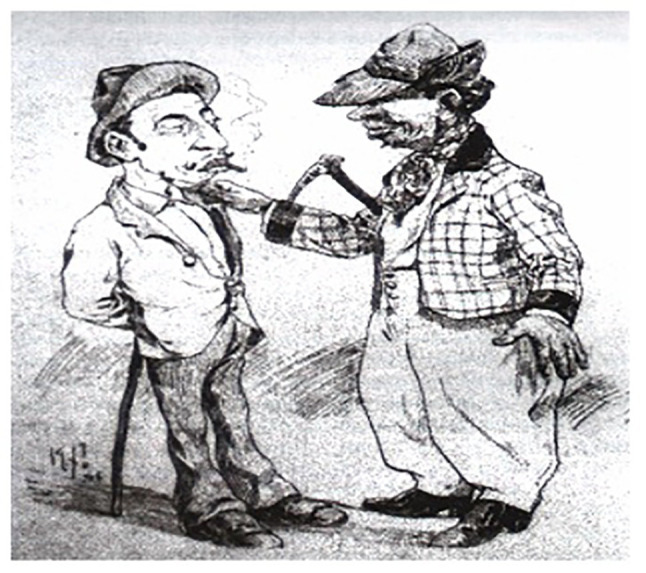
Sifting.

**Figure 3:**
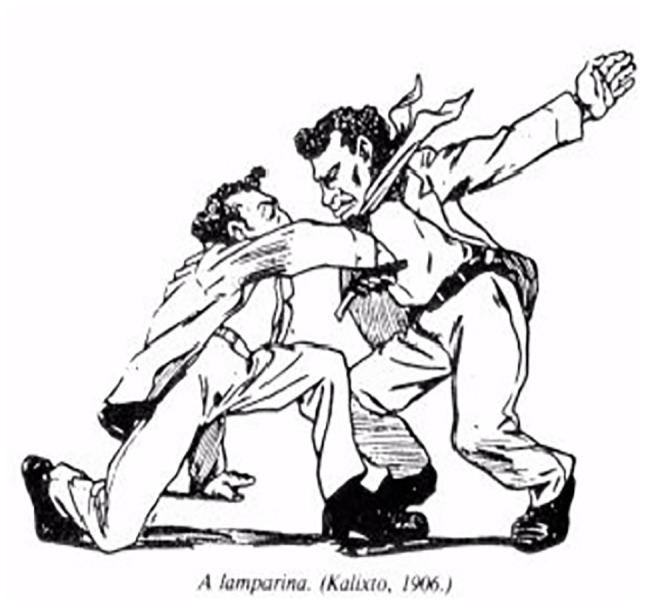
The oil lamp.

Another element that confirms the lives of *carioca capoeiras* is also recorded by Calixto Cordeiro and Álvaro Martins (quoted in Santos, no date), when it represents the defense weapons used by *capoeiras*, practitioners or not of this Brazilian fight; many of the descriptions given by Araújo (1997) are confirmed, in which the stick gains relevance,^13^ present in the image The Sifting (Figure 1), which should never exceed 50 cm of canes (Figure 2) depicted in Types and Uniforms, or the knife (Figure 3; Freyre, 2000), shown in the illustration The Oil Lamp, numerous times portrayed in manuscripts and social and police chronicles of the 19th century, a defense weapon historically linked to individuals covered by the nickname *capoeiras*, and thus, allowing to remain unchanged, by means of this iconographic representation, the myth of the close connection between the knife/Capoeira.

As mentioned above, these images are invaluable for the understanding of the history of Capoeira and of one of its fundamental elements, its movements, and strikes used by practitioners in the 19th century, given the scarcity of pictorial records only represented by the drawings of Rugendas, Earle and Christiano Jr. during that century.

This iconographic record allows us to get closer to the written records of those cornerstones submitted by ODC (1907), Burlamaqui (1928), Edmundo (1932) and Moraes Filho (1999), and thus reconstruct the framework of movements and strikes of this expression over time, either by consideration of its expressive form of a corporal character or by the nominal identifications assigned to them in different periods, regions, or styles of Capoeira.

**Figure 4:**
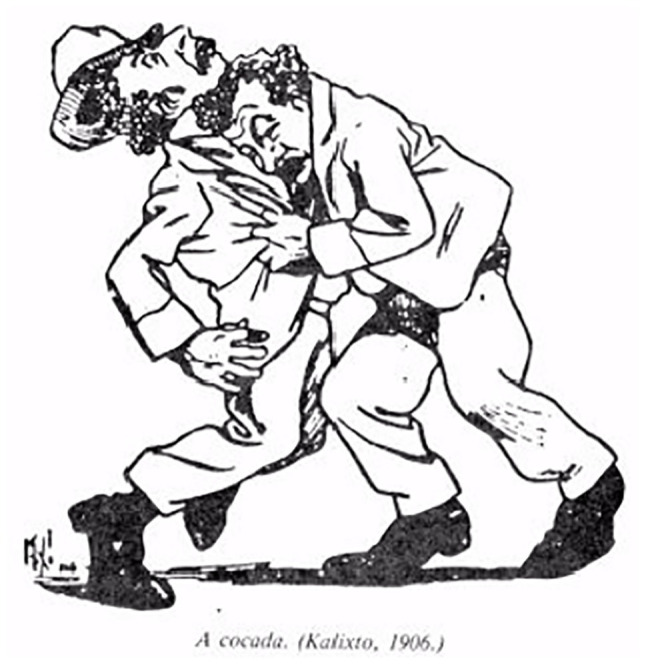
The coconut strike.

Considering the approaches made by Araújo and Jaqueira (2010) on the study of strikes and movements within Capoeira, the movements represented by the caricaturist and nominally identified as The Sifting, The Oil Lamp, The Coconut Strike had other names for more than three quarters of the 19th century, *negacear* (Figure 1), *tapona* (Figure 3; ODC, 1907; Edmundo, 1932; Araújo and Jaqueira, 2008), and *cabeçada* (Figure 4), some of them described in contemporary dictionaries (Novo Aurélio Século XXI: O Dicionário da Língua Portuguesa, 2000) with different meanings from those presented by the description of Lima Campos.

Some of these names were widely described in documentary^14^ and bibliographic (Edmundo, 1932; Moraes Filho, 1999; Freyre, n.d.) records that portrayed the presence of the colonial *capoeiras*, practitioners or not of the Brazilian fight, in which for the word *cabeçada* (head strike) there were several others, depending on the specific parts of the body to hit, like chin; chest; belly; in the face, and also the word *chifrada* (strike with a horn).^15^

Although we find many allusions to the lack of documents about Capoeira in the state of Bahia, we are able to prove the presence of *cabeçada* in the 80s of the 19th century in this state^16^ and the beginning of the 20th century^17^ in the descriptions below:

*On the night of February 22, 1883, the soldier José Raimundo de Souza, standing patrol in the Baixa dos Sapateiros, went to arrest the stevedore Celestino, author of a major conflict on that street, from whom he received a ‘cabeçada’ that caused him almost instant death* (Araújo, 1949).

*The soldier, retreating a bit, pulled the saber to frighten the aggressor. He could do nothing, however, because (…) he, skilfully, in a Capoeira step, dodged the blows, finally hitting Aristide with a great ‘cabeçada’, making him fall in an agonising fashion* (Um crime passional e uma cabeçada que mata, 1916).

**Figure 5:**
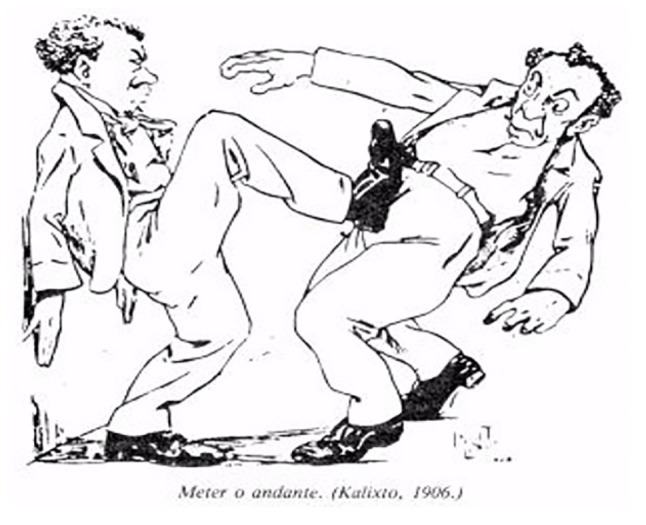
The mower.

The movement The shim or Tripping (Figure 5), besides being present in Edmundo (1932), are also highlighted in ODC (1907) and Moraes Filho (1999) with the same name, although, under this double reference, it should be noted that they are two separate blows in the context of this fight, and so recognized by other writers who referred to the presence of these movements as two different ones. The Burlamaqui (1928) also considers the shim effectively a trip, but with a different form of expression from the one presented by Calixto Cordeiro in his image, which he identifies as the strike known as The Falling from the Slope.

In short, a strike called shim permeated the context of Rio Capoeira in the 19th century and the first two decades of the 20th century, fitting as a kind of trip, without, however, asserting itself as identical to the trip typified this caricature, only converging on the understanding that they are different movements but both are applied with the leg or foot to knock the opponent.

The trip configured in this cartoon is presented with the characteristic already identified by several authors who described some of the movements and strikes of Capoeira in the late 19th century and early 20th century, as the mower trip (Burlamaqui, 1928) or hunter trip (Moraes Filho, 1999) or the trip or *rabo de arraia* in three stages (Pederneiras, 1926), Plácido de Abreu being (1886) the first author to particularize and describe one of the ways to implement the many trips that we know today.

There followed several descriptions of this type of strike in the works of ODC (1907) and Burlamaqui (1928) as those that showed more characteristics of this type of attack movement, typifying and nominating them in relation to their temporality (ancient and modern), to the planes (transversal, sagittal, frontal) with the hands or feet, and to the orientation according to the performed physical action (from the front, back, side, outside, inside).

**Figure 6:**
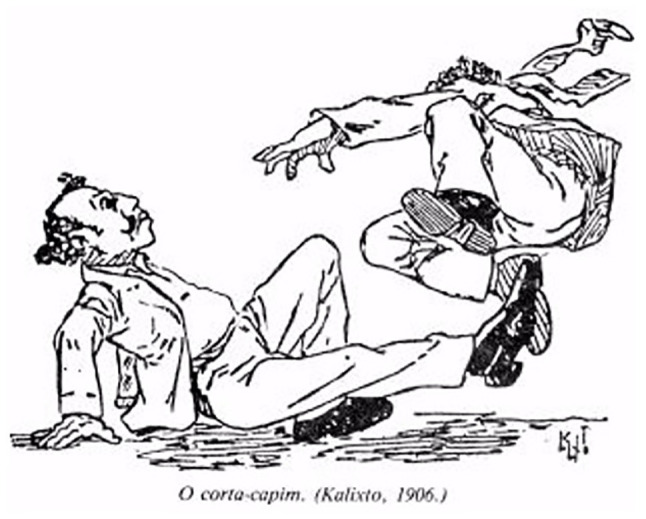
Tuck the move.

As to the term Tuck the Move (Figure 6), we believe this identification configures a slang feature in this context, as no citation by the chroniclers of the time or any similar primary source reference are not found historically; only one particular occurrence stands out in the iconography of Earle (quoted in James, 1955) – a movement with a body expression similar to that indicated by Calixto Cordeiro in his caricature but without any assigned name.

We found a similar physical expressiveness in the movements identified as plate or abutment (Figure 6; ODC, 1907; Burlamaqui, 1928) and already referenced in numerous works by the authors mentioned in this text, even with no specific designation, and that, at present, conform to the nominal identification of blessing (Figure 6; Mestre Bimba, campeão na capoeira desafia todos os luctadores bahianos, 1936), or even with these names in many existing Capoeira groups.

In the years that followed the production of these images, many were the authors who have registered, through their literary productions on the fight of Capoeira, the description and identification of these movements with different or identical names, by selecting only these names for a strike with this feature according to the identity of the group with a view to their social or stylistic differentiation, by linguistic regionalism, or even by individual issues of the creators of the new groups of this Brazilian fight expression.

## Discussion

In concluding this analysis of the caricatures of Calixto Cordeiro, we consider the relevance of your work to the understanding of various inherent aspects to this Brazilian fight, which features the presentation not only of its own language, of specific costumes of the *capoeiras*, of defense weapons that always accompanied them but also, and above all, of the identification of some movements belonging to the gestural framework of Capoeira, very poor in descriptive and iconographic records of any kind up to that moment.

In deciding on a multi-method process for this analysis, and in particular on the assumptions indicated by Panofsky, we consider that this methodological choice proved to be right for this type of analysis, initially iconographic, but that, from the confrontation with the data and facts contained in the different sources consulted on Capoeira, relevant insights were provided on the different elements alluded to in this article and on the cultural history of this Brazilian expression.

Thus, the results derived from the analysis of the form expressed in Calixto’s images, when confronted with the different sources consulted and providing information of a bodily, symbolic, linguistic, and indumentary nature, contributed to consider that an iconological study had taken place, because it did not focus only on the analysis of form and content but also because it enabled interpretations that enriched the knowledge of aspects inherent to Capoeira in the sociocultural context during the 19th century and the early 20th century.

The iconography analyzed greatly contributes to the historical reconstruction of this body expression over time, safeguarding the regional peculiarities, well established already in the documentary evidence discovered so far and disclosed in some recent works on this genuinely Brazilian cultural manifestation.

## Author Contributions

All authors listed have made a substantial, direct and intellectual contribution to the work, and approved it for publication.

### Conflict of Interest

The authors declare that the research was conducted in the absence of any commercial or financial relationships that could be construed as a potential conflict of interest.

## Footnotes

^1^According to Paulo Araújo, the individuals belonging to marginal groups, also called *capoeiras*, are those considered outside the legal framework of society, slaves, local or Guinean blacks, mamelucos, and white maladjusted who, making use of the spaces and ways of community life found there, promoted a form of struggle against the state system that oppressed them. *Abordagens sócio-antropológicas da Luta/Jogo da Capoeira (Maia: Publismai, 1997), p. 79.*

^2^Orser Jr., Introdução à Arqueologia Histórica, 57.

^3^Lima Campos. A Capoeira. Revista Kosmos, Rio de Janeiro, Anno III, n˚ 3, 1906.

^4^Paulo Araújo and Ana Rosa Jaqueira, *Do jogo das imagens às imagens do jogo. Nuances de interpretação iconográfica sobre a Capoeira* (Coimbra: Centro de Estudos Biocinéticos, 2008), 109.

^5^Jorge de Alarcão, *A escrita do tempo e a sua verdade* (Coimbra: Quarteto Editora, 2000), 124.

^6^Alarcão, A escrita do tempo e a sua verdade, 130.

^7^Eduardo França Paiva, *História and imagens* (Belo Horizonte: Autêntica Editora, 2002), 19.

^8^Paiva, História & imagens, 31.

^9^Lima Campos, “A Capoeira”, *Revista Kosmos*: Anno III, n. 3 (1906): 56–59.

^10^According to Paulo Araújo, groups of *capoeiras* are forms of collective expressions of individuals known as *capoeiras*, often identified as gangs, groups, hoards, gatherings, and, finally, by *malta* (guys), which may be the most significant word to interpret the group events that sought to disrupt the established social order, be they effective practitioners of the Capoeira fight or not. *Abordagens sócio-antropológicas da Luta/Jogo da Capoeira* (Maia: Publismai, 1997), 161–162.

^11^Many authors who have studied this collective phenomenon, when referring to the organizational structure of the capoeira guys, portray them as an autonomous and hierarchical body, from the chiefs and sub-chiefs up to the *carrapetas* (lowest orders), in which all the members had their duties and functions fully encoded. Araújo, Abordagens sócio-antropológicas da Luta/Jogo da Capoeira, 174.

^12^Alexandre José Mello Moraes Filho. Festas e tradições populares do Brasil (Belo Horizonte/Rio de Janeiro: Editora Itatiaia, 1999), 258 and 262.

^13^Brasil, Codex 403 Vol. II. 01/03/1820. Arquivo Nacional.

^14^See Note 12.

^15^Plácido Abreu, 1894, 95.

^16^Oséias Moreira de Araújo. Notícias sobre a Polícia Militar da Bahia no Século XIX (Salvador: Imprensa Oficial da Bahia, 1949) 24.

^17^“Um crime passional e uma cabeçada que mata.” A Tarde (Salvador, 18 December 1916) 3.
